# Ocean acidification effects on *in situ* coral reef metabolism

**DOI:** 10.1038/s41598-019-48407-7

**Published:** 2019-08-19

**Authors:** Steve S. Doo, Peter J. Edmunds, Robert C. Carpenter

**Affiliations:** Department of Biology, California State University, Northridge, United States

**Keywords:** Marine biology, Carbon cycle, Ecosystem ecology

## Abstract

The Anthropocene climate has largely been defined by a rapid increase in atmospheric CO_2,_ causing global climate change (warming) and ocean acidification (OA, a reduction in oceanic pH). OA is of particular concern for coral reefs, as the associated reduction in carbonate ion availability impairs biogenic calcification and promotes dissolution of carbonate substrata. While these trends ultimately affect ecosystem calcification, scaling experimental analyses of the response of organisms to OA to consider the response of ecosystems to OA has proved difficult. The benchmark of ecosystem-level experiments to study the effects of OA is provided through Free Ocean CO_2_ Enrichment (FOCE), which we use in the present analyses for a 21-d experiment on the back reef of Mo’orea, French Polynesia. Two natural coral reef communities were incubated *in situ*, with one exposed to ambient pCO_2_ (393 µatm), and one to high pCO_2_ (949 µatm). Our results show a decrease in 24-h net community calcification (NCC) under high pCO_2_, and a reduction in nighttime NCC that attenuated and eventually reversed over 21-d. This effect was not observed in daytime NCC, and it occurred without any effect of high pCO_2_ on net community production (NCP). These results contribute to previous studies on ecosystem-level responses of coral reefs to the OA conditions projected for the end of the century, and they highlight potential attenuation of high pCO_2_ effects on nighttime net community calcification.

## Introduction

Tropical coral reefs provide coastal protection, livelihoods, and food to millions of people^1^. However, these goods and services are threatened by rising concentrations of atmospheric carbon dioxide (CO_2_) from anthropogenic sources^2^. A result of rising atmospheric CO_2_ concentrations is global climate change (GCC), which for tropical coral reefs is causing profound modifications in benthic community structure and function through thermally-driven mass bleaching events^3,4^. Ocean acidification (OA) is a process through which dissolution of CO_2_ into seawater lowers the calcium carbonate saturation state of seawater (Ω)^5^. In the marine environment, OA is expected to negatively affect organisms which produce calcareous shells and skeletons more than non-calcifying taxa^6–8^. These effects will be experienced acutely by coral reefs, where the physical structure of the calcareous substratum is built by scleractinian corals and calcified algae^7^. OA is predicted to severely impact the underlying biogenic substrata of coral reefs through increased dissolution in response to acidifying seawater conditions^9,10^. Field-based observations in natural CO_2_ seeps have shown decreased coral cover, diversity, and function in response to acidified conditions^11^. However, changes in carbonate chemistry across large spatial scales can interact with local effects to modulate processes that contribute to reef carbonate production (e.g. calcification and bioerosion)^12^. A common theme across papers addressing this topic is that OA is threatening the underlying biogenic physical structure of reefs and, therefore, understanding these effects has become a central objective of coral reef research in the 21^st^ Century^13^.

Depression of Net Ecosystem Calcification (NEC), the balance of gross calcification and gross dissolution, has been predicted for coral reefs in response to OA and warming^14,15^. However, the complexities of understanding scaling effects^16^ as well as feedback loops between the inorganic substrata and living biota^14^ have resulted in difficulties attributing natural variation in NCC rates, and limited the ability to detect and attribute OA impacts on coral reefs. Laboratory-based studies with constructed communities have shown OA will increase dissolution of coral reefs^17,18^, as well as decrease their functionality through an alteration of the relationship between photosynthesis and calcification^19^.

While the impacts of OA on corals, other reef organisms, and reef communities are relatively well known^7^, *in situ* studies testing community-level responses are needed to predict how marine biota will respond to a changing climate^20^. Experiments conducted *in situ*, including those employing a Free Ocean CO_2_ Enrichment (FOCE) approach, are the benchmark for ecological relevance for determining OA effects on ecosystem function^20^ yet there are substantial challenges to implementing FOCE approaches on coral reefs. There are great benefits to addressing these challenges, however, as FOCE experiments embrace the natural complexity of ecosystems in terms of evaluating their response to OA, notably through emergent properties of multiple taxa interacting in a chemically and physically complex environment. To date, there have been two projects in which the effects of elevated CO_2_ have been empirically tested *in situ* on coral reefs. The first, by Albright *et al*. 2018, measured NEC of the back reef community at One Tree Islands, Australia, in response to short-term pulses of acidified seawater over multiple days^15^. A second project was conducted on Heron Island, Australia, in which deployment of technology described as a “coral proto-FOCE” (cp-FOCE) showed decreases in coral calcification^21^, and alteration in the boron isotopic composition of coral skeletons^22^. Here, we significantly expand on these previous experiments by conducting a FOCE experiment to test the effects of predicted end-of-century OA conditions on NCC and Net Community Production (NCP) of a natural back reef community in Mo’orea, French Polynesia. Our results show how the ecosystem function of coral reefs (NCC and NCP) will be altered by OA during the day and night, resulting in improved accuracy of predictions of the effects of OA on coral reef metabolism.

## Results and Discussion

### Efficacy of treatment conditions

**Figure 1:**
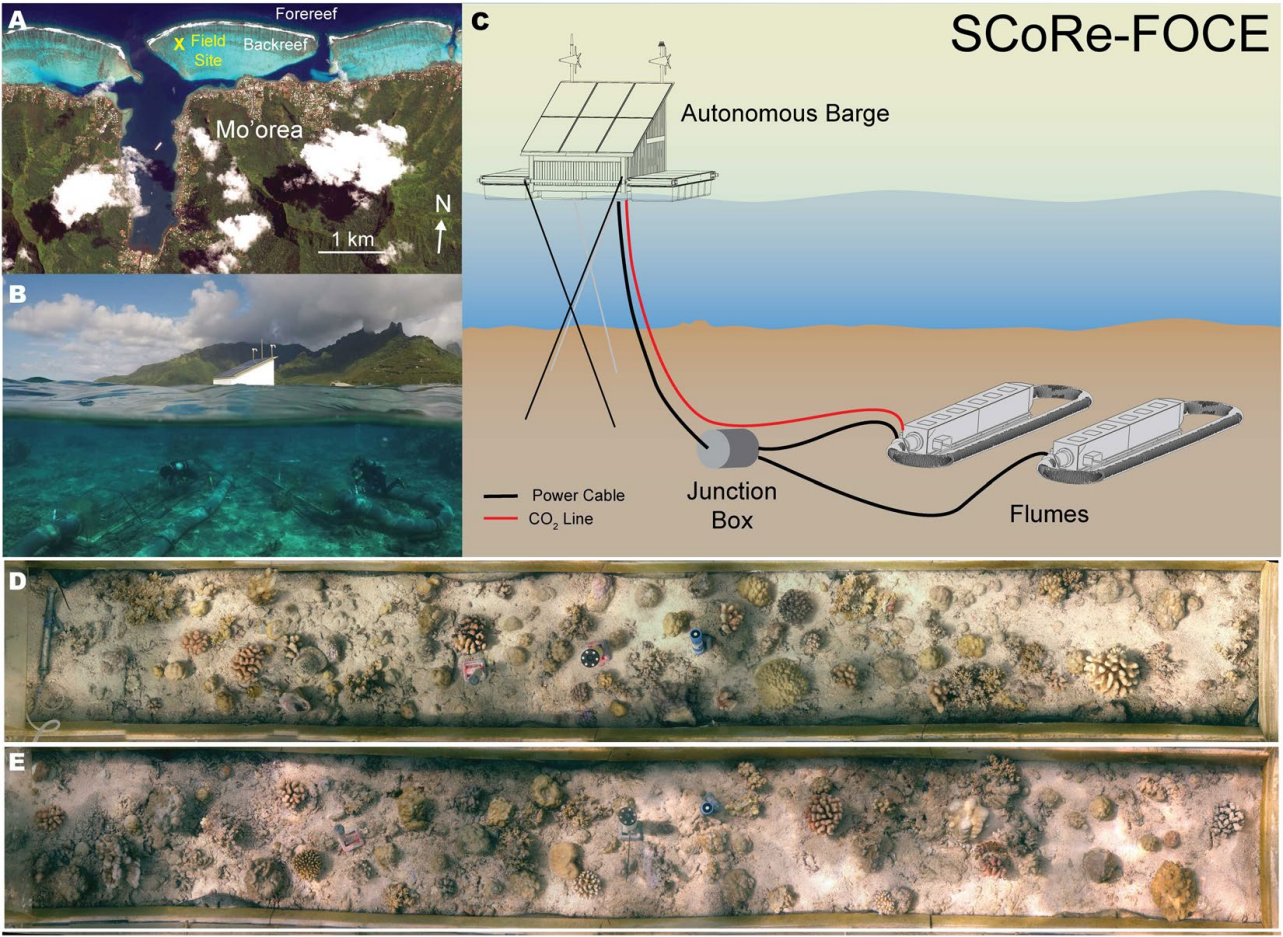
(**A**) Aerial WorldView-2 image (Copyright 2018, Digital Globe, Inc.) of the north shore of Mo’orea, French Polynesia, with the study site shown with a yellow cross. (**B**) Photograph of the *in situ* flumes with divers sampling seawater, and the autonomous floating platform above and the north shore of Mo’orea in the background. (**C**) Schematic of the study site showing the layout of the experiment including the flumes and floating platform. Pre-selected undisturbed plots of reef were used in this study for the community incubated in high CO_2_ (**D**) and ambient conditions (**E**).

Our study presents the first community metabolism results of the deployment of a FOCE experiment on a shallow, back reef community, and it describes the response of this community to high pCO_2_ under ecologically relevant environmental conditions^23^. Our experiment was conducted on two plots of coral reef (5.00 × 0.55 m) (Fig. 1A) that were similar to one another in benthic community composition at the start of the experiment (the cover of corals and crustose coralline algae [CCA] differed <4% between the plots; Fig. S1), and were ~1 km from the shore. Acrylic flumes (1.5-cm wall thickness with UV-transparent tops) without floors were sealed over each plot to allow for measurements of metabolism (Fig. 1B), and the manipulation of seawater pCO_2_ over 21 days (2–23 May 2018). Unidirectional flow speeds within the flumes were maintained at ~14 cm s^−1^ using motor-driven propellers, and were similar to average, long-term flow speeds recorded on the back reef (Table S1). Using an autonomous CO_2_ dosing system deployed on a floating platform adjacent to the flumes, ambient or elevated pCO_2_ conditions were created for each community (Fig. 1), which were assigned randomly to one of the two flumes. Elevated pCO_2_ was maintained by CO_2_ gas-enrichment to *in situ* seawater, with pCO_2_ regulated through negative feedback provided by a pH electrode fitted to the flume (Fig. 1C; see methods).

Our experiment contrasted the effects of ambient pCO_2_ (393 ± 4 µatm, n = 950) and elevated pCO_2_ targeted at ~1000 µatm (949 ± 7 µatm, n = 950; corresponding to a seawater pH_T_ of ~7.72; Fig. S2). These conditions reflect global atmospheric CO_2_ concentrations that equilibrate with seawater to create Ω_arag_ of 3.98 and 2.09, respectively (Fig. S3,B). The elevated pCO_2_ is predicted to occur by the end of this century under a pessimistic projection (RCP 8.5) of human actions to control CO_2_ emissions^24^. NCC and NCP measurements were made over three consecutive days arranged into four sampling blocks equally spread over the 21-d experiment (i.e.,12 incubation days; see methods). Each day of measurements consisted of determinations in the morning, mid-day, afternoon, and once at night.

### Effects of ocean acidification on *in situ* reef metabolism

**Figure 2:**
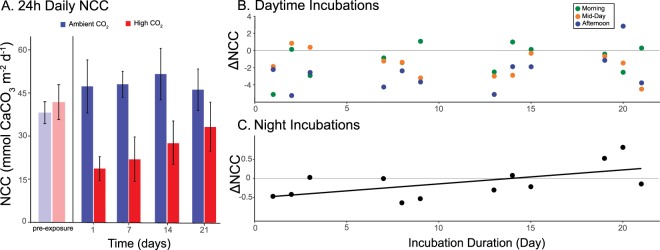
Metabolism of back reef communities incubated under ambient and elevated pCO_2_. (**A)** Mean (±s.e.m., n = 3 d) change in 24-h NCC of communities exposed to ambient (393 µatm; blue) or high (949 µatm; red) pCO_2_ over 21-d. NCC was measured three times during the day (average within day) and once at night over four blocks of three days each, beginning on day 1 (initial values) and ending on day 21 (final). Pre-exposure values show NCC in flumes before CO_2_ dosing began. (**B)** Scatterplot showing the difference in daytime (three measures [colors] n = 36) NCC between ambient and high CO_2_ (ΔNCC, mmol CaCO_3_ m^−2^ h^−1^) as a function of days of the experiment (n = 36), with no linear relationship between the two (P = 0.295, no line shown) (**C)** Scatterplot showing how the difference in nighttime NCC between ambient and high CO_2_ (ΔNCC) as a function of days of the experiment (n = 12), with the line showing the Model I linear relationship (P = 0.040).

Following initiation of the experiment, 24-h NCC was depressed by 47% within the first day of the high pCO_2_ treatment, and remained consistently depressed relative to 24-h NCC under ambient pCO_2_ (Fig. 2A). This effect corresponded to a 25% decrease in NCC per unit Ω_aragonite_ decline, which is similar to the effect size reported in a previous meta-analysis of the sensitivity of reef corals to OA^7^. Overall, there was a 49% reduction in daytime NCC under high versus ambient pCO_2_ (Fig. 2B), which corresponds to a 26% reduction in NCC per unit Ω_aragonite_ decrease. At One Tree Island, Australia, NCC of a lagoon reef was reduced 43% per unit Ω_aragonite_ reduction during the day, with this effect revealed through an experimental decrease in pH of ~0.14 from ambient (0.7 Ω_aragonite_ reduction)^15^. In contrast, our study employed a greater decrease of pH (i.e., ~0.38), and a reduction in Ω_aragonite_ of 1.89 between treatments, suggesting the decrease of NCC is not a linear function of Ω. On One Tree Reef, there was a higher cover of crustose coralline algae (CCA) (26%), and lower coral cover (12%) compared to the communities in our study (12.2% and 21.9%; CCA and coral, respectively; Fig. S1)^15^. This contrast in community structure at One Tree Reef (as described in^15^) versus the back reef of Mo’orea could indicate that the coral reef community studied at One Tree Island was more sensitive to declining seawater pH than the present coral reef community studied in Mo’orea^10^.

On the first day that the treatments were established in the present study, nighttime NCC of the coral reef community maintained under high pCO_2_ was depressed by 74% relative to ambient conditions. However, the magnitude of the pCO_2_-mediated depression of nighttime NCC attenuated over time, and after ~14 days the effect of pCO_2_ reversed, such that nighttime NCC in the elevated pCO_2_ treatment was ~1% higher than that under ambient conditions. After 21 days, nighttime NCC at elevated pCO_2_ was 27% greater than nighttime NCC at ambient pCO_2_ (Fig. 2C). Coral reefs, and in particular, the carbonate sediments packed within their framework, are predicted to transition to net dissolution under RCP 8.5, which was used in the present analysis to scale the pCO_2_ treatment applied^9^. This effect potentially is attributed to the role of OA in accelerating the dissolution of CaCO_3_ produced by calcareous organisms (e.g., high Mg-calcite producing CCAs vs aragonite producing corals^21^). The overall effect would result in a positive slope of NCC differences between ambient and high pCO_2_, shown as less reef dissolution over the 21-d experiment. While recent work has shown little acclimatization potential of reef calcifiers to OA^25^, the attenuation of the initially negative effect of high pCO_2_ on nighttime NCC of a shallow coral reef suggests these communities acquire some resistance to the negative effects of OA on nighttime NCC. The proximal mechanism(s) underlying this response are unknown, but potentially could include a rapid physiological acclimatization of calcification in organisms to high pCO_2_ as seen in polychaetes transplanted to CO_2_ vents^26^. While we did not observe a change in coral cover during the experiment, biological feedback loops caused by changes in relative composition of the microbial benthic community should be considered as a potential mechanism mediating the change in nighttime NCC^27^. Investigation into this mechanism might benefit from a further understanding of the relationship between biogenic processes that affect NCC and geochemical changes (e.g., mineral composition) within the pore water of the reef framework, as this interaction has been shown to be susceptible to OA^28^. Further work is required to explore these possibilities, and to determine whether the effects observed for nighttime NCC might also affect daytime NCC and/or 24-h NCC (Fig. S4).

Our study provides the first experimental results of an *in situ* effect of OA on NCP (Fig. S4,A), in which NCP was depressed by 24% at high pCO_2_ compared to ambient pCO_2_ when averaged across the entire experiment (Fig. S5,A, S. Table 2). NCP during the second incubation (Days 7–9) was significantly higher than during the initial incubation period (Fig. S5,A, S. Table S2), an effect likely due to increased light levels experienced during this time (Fig. S3,D)^19,29^. Respiration (nighttime oxygen flux) was 41% higher in the community exposed to ambient pCO_2_ versus high pCO_2_ during the first 3-d incubation (Fig. S5,B; S. Table 2E), and community respiration increased by an average of 66% in both communities over the experiment (Fig. S5,B; S. Table 2E). Decreased NCP of a coral reef community exposed to OA differs from the null result recorded for the same response variable when a back reef community from Mo’orea was exposed to high pCO_2_ (1146 µatm) for four months^19^. While CCA cover increased in both treatment groups in the present study, a greater proportional increase in algal turf cover and cyanobacteria occurred in the reef community maintained at high versus ambient CO_2_ (Fig. S1). This outcome suggests fast settling turf/cyanobacteria may drive the changes observed for NCP and R.

### Functional changes of reef communities in response to OA

**Figure 3:**
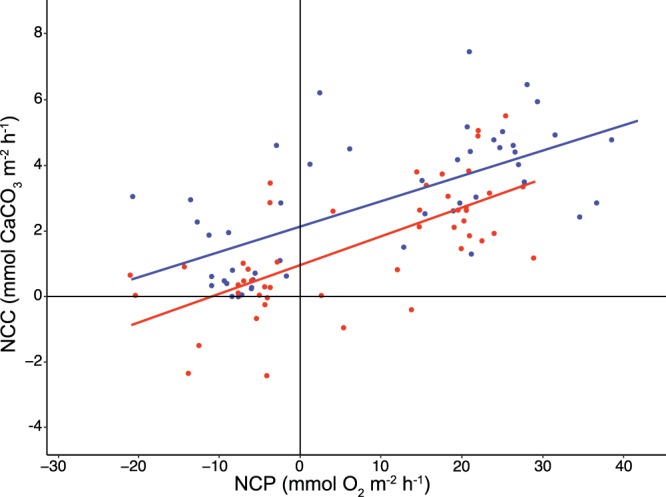
Model II regressions of NCP against NCC describing the relationships between primary production and calcification over all sampling times. All measurements corresponding to each time point (see methods) of ambient (393 µatm; blue) and high (949 µatm; red) pCO_2_ were regressed over the 21-d period.

Functional shifts in coral reef communities that alter the ratio of primary production to calcification are reflected in the slope of the NCP-NCC relationship, with a reduction in this relationship representing degradation in reef function, generally resulting from a shift in the dominant benthic community structure from calcifying organisms to algal-dominated communities^30,31^. In the present study, we did not observe any change in the NCP-NCC slope under high pCO_2_, (0.077 ± 0.027 for ambient conditions; 0.086 ± 0.028 mmol O_2_ m^−2^ h^−1^ for high CO_2_; slope ± 95% CI; *χ*^2^ = 0.142, p = 0.706; Fig. 3). Previous field studies have documented that a change in the NCP-NCC slope reflects a change in benthic community composition^31,32^, although the communities in both of the present treatments did not change significantly over time (Fig. S1,A). A previous study by Page *et al*. (2016) comparing the response of mixed vs homogenous benthic community composition representing reefs found in Kaneohe Bay (Oahu, Hawai’i) to acidified conditions showed similar results to those reported here in which the NCP-NCC slope did not change significantly as a result of increased CO_2_^29^. However, in the present study, we observed that the intercept was reduced by 58% for the community incubated under high CO_2_ (0.927 ± 0.402) versus ambient CO_2_ (2.129 ± 0.478) (both ± 95% CI; *W*_*T*_ = 16.913, p < 0.001). While previous studies of reef community metabolism have not reported alteration of the intercept of the NCP-NCC relationship as a metric of change in metabolic function, the alterations of elevation between treatment groups while maintaining similar slopes seen in our study indicate that a shift in overall community function occurred in the community exposed to high CO_2_, where increased rates of NCP, are required to achieve similar rates of NCC^19^. For the reef communities in the present experiment, these changes likely are caused by a combination of decreases in the calcification rates of individual organisms and an increase in overall dissolution under high pCO_2_^10^.

Global climate change is causing large declines in coral cover on reefs worldwide, particularly through the effects of mass bleaching^3^. Together with experimental analyses of the effects of global climate change and OA on corals, the rapid declines in coral cover have fueled concerns that coral reefs may not persist as calcified entities beyond the end of the current century^15^. The benthic community structure and ecological functions of coral reefs are tied intrinsically to the success of their ecosystem engineers, scleractinian corals and calcified algae, and these taxa already have been impacted by a wide variety of local disturbances that act in concert with global anthropogenic effects which cause a change in ecosystem functioning^33^. To date, predictions of how coral reefs will respond to OA have been based largely on species-level experiments testing the sensitivity of coral calcification to high pCO_2_^16^. Our study highlights that the “organism approach” cannot capture the functional complexity arising from multiple organisms operating in concert in a natural environment subject to routine variation in select environmental conditions (e.g., light and temperature). An important implication of this outcome is that the whole coral reef community is more than the sum of its parts with regard to its response to OA. The present study highlights the importance of these emergent properties through *in situ* analysis of an undisturbed coral reef community. Our results suggest that these communities have the potential for adjustments to partially alleviate the negative consequences of OA on NCC. While this outcome might attenuate the risks of OA-related dissolution of the carbonate framework supporting most corals reefs^10^, the absence of a comparable effect on daytime NCC underscores that a further understanding of mechanisms that drive changes in NCC are needed.

## Methods

Our study was conducted on the back reef of the north shore of Mo’orea, French Polynesia, which is a high volcanic island in the South Pacific. A custom-designed Shallow Coral Reef Free Ocean CO_2_ Enrichment (SCoRe-FOCE) system^23^ was used to enrich pCO_2_ to levels projected for 2100 under representative concentration pathway (RCP) 8.5, which assumes a “business as usual” scenario with regards to anthropogenic emission of CO_2_^24^. In February 2016, two plots were identified in the back reef, each 5.00 m long by 0.55 m wide, and they were selected to have a benthic community similar to this back reef in 2007^18^, which consisted of 22% coral, 12% CCA, 23% turf algae, and 43% sand and rubble. Although this community structure differed from that occurring in 2018 when the experiment was conducted, our goal was to explore the response of an average back reef community, which is well represented by the state of the back reef in Mo’orea in 2007. When the study plots first were chosen, coral cover had declined compared to 2007 and, therefore, the community structure was augmented through transplantation of a few coral colonies from the adjacent reef to the study plots. Transplantations were completed >6 months prior to initiation of the incubations.

Two-weeks before the start of the experiment (15 April 2018), two, 1.5-cm thick clear acrylic flumes (5.0 × 0.55 × 0.55 m (length × width × height) with UV-transparent tops were secured to fiberglass rails previously attached to the reef at the perimeter of each study plot. The internal volumes of each flume and the return-section was ~2,300 L. The flume was secured to the reef with stainless steel threaded rods drilled and epoxied into the carbonate substratum, and was sealed to the reef using rubber tubing inflated with water and placed between the supporting rails and the reef surface. Rubber matting extending 30 cm from the rails and weighted using 10-kg sand bags augmented the seal. An indicator dye (Rhodamine B, Matheson Coleman & Bell) was injected (~1 mg L^−1^) into the closed flumes at the start and end of the experiment to evaluate the efficacy of the seal to the reef, and visual inspection was used to detect leaks; none were visible during ~2 h trials.

### CO_2_ enrichment

The flumes were powered and controlled through an umbilical cable connecting them to a nearby floating platform fitted with solar panels, wind turbines, and batteries (Fig. 1). CO_2_ dosing to the flume was controlled through an Apex Aquacontroller (Neptune Systems), connected to an Atlas Scientific pH probe (ENV-40-pH), which controlled a solenoid (McMaster-Carr Model 5077T141) that injected CO_2_ gas into the flume from a 60-L gas cylinder on the floating platform. pH probes that controlled the autonomous dosing system were calibrated against pH values every 3 days using the m-cresol dye method (SOB 6 b Dickson^34^). Adjustments were made daily to maintain CO_2_ conditions within the flume (Fig. S2). The seawater in the ambient flow was not manipulated with respect to CO_2_. The pH of the elevated pCO_2_ flume was set to a daytime target (06:00–18:00 h) of 1000 µatm pCO_2_ (~7.70 pH_Total_ units), and the system was programed to decrease by 0.1 pH units (i.e., ~1300 µatm pCO_2_), at night (18:00–06:00 h) to mimic *in situ* diel oscillations of pCO_2_ recorded on the backreef of Mo’orea^35^ (Fig. S2). A SeaFET pH sensor (Durafet® pH sensor) was deployed in each flume to continually record seawater pH, and these instruments were calibrated every 3 days^36^. Calculation of carbonate chemistry parameters were performed in CO2SYSv2.1 using pH_Total_ and A_T_ as the two input parameters.

### Incubation parameters and calculation of community metabolism

During the experiment, each flume was flushed with ~200 L h^−1^ ambient seawater that was pumped from the reef within 50 m of the study plots. Sampling for NCC and NCP was performed on days 1–3, 7–9, 13–15, and 19–21 of the 21–d experiment (Fig. S3). Each day of sampling consisted of three incubations during the day (06:30 to 09:30 h, 10:00 to 14:00 h, and 14:30 to 17:30 h) and one incubation that extended over the night (18:00 to 06:00 h). During the measurements of metabolism, flushing of the flumes with seawater was halted, but CO_2_ treatments and flow conditions were maintained. To prevent hyperoxia and hypoxia in the flume during closed-circuit operation, seawater from the surrounding reef was pumped into the flume for 30 min in between each incubation to replace ~25% of the volume. For the flume maintained at high pCO_2_, flushing with ambient seawater between incubations decreased pCO_2_ to ambient levels. However, following cessation of flushing and prior to the next incubation, treatment conditions were restored to target pCO_2_ values (~1000 µatm) within 15–30 min.

For each incubation, samples of seawater were collected at the beginning and end of each incubation to quantify seawater carbonate chemistry. Samples were drawn from the flume using a 60-mL syringe that was attached to a vinyl tube fitted with a shut off valve. Samples were transported immediately to the shore lab, where salinity was measured using a Thermo Scientific Orion Star A212 conductivity meter, then potentiometrically titrated following standard operating procedures (SOP 3b of Dickson *et al*. [2007]) using an automatic titrator (Mettler-Toledo T50) fitted with a Rondolino-sample carousel (Mettler-Toledo). The titrator was fitted with a Mettler pH probe (DGi-115) that was operated with certified HCl (Batch A13 Dickson Laboratory). Certified reference material (Dickson CRM Batch #138) was used to evaluate the accuracy of the total alkalinity (A_T_) measurements (SOP 3b^34^).

NCC was quantified using the alkalinity anomaly method^37^ where $\,{\rm{\Delta }}{A}_{Tfinal-initial}$ (µmol kg^−1^) was calculated from the difference between A_T_ in final and initial salinity-normalized water samples from each flume:

$$NCC=\frac{-({\rm{\Delta }}{A}_{Tfinal-initial})}{2t\times SA}\times \rho V$$

where *t* is time (h), *SA* is the planar surface area of the reef enclosed by the working section of the flumes (m^2^), *ρ* is the density of seawater (1.023 kg L^−1^ and calculated from average salinity, and temperature from each daily measurement), and *V* is the internal volume (L) of the flume (including the return sections).

NCP was measured from the rate of change of O_2_ concentration as a function of time, with dissolved O_2_ concentrations measured using MiniDOT O_2_ sensors (Precision Measurement Engineering, Inc.) with one sensor in each flume. We chose to measure O_2_ flux as opposed to measuring DIC changes to maintain treatment conditions through CO_2_ dosing. Rates of change were determined using least squares linear regression of O_2_ concentration (mmol L^−1^) against time (h) (final units of mmol O_2_ m^−2^ h^−1^). All O_2_ sensors were within factory calibration, which is stable for ~1 year.

NCP was calculated from O_2_ fluxes where *DO* is the change in O_2_ concentration (mg L^−1^), molar mass of O_2_ (32 g mol^−1^), *SA* is benthic surface area enclosed in the flumes (2.5 m^2^), *t* is incubation duration (h), and *V* is the volume of the flume (L).

$$NCP=\frac{DO\,}{molar\,mass\,O2\times SA\times t}\times V$$

### Data analysis

#### NCC and NCP

To calculate time-integrated values for NCC and NCP (calculated over 06:30–09:30 h, 10:00–14:00 h, 14:30–17:30 h, and 18:00–06:00 h), hourly values were integrated over each 3–12-h incubation period and summed within each day to obtain 24-h values. For 12-hr daytime values, integrated 3 h incubation periods were summed from 06:00–18:00 h, and for 12-hr nighttime values, data were integrated from 18:00–06:00 h

#### Analysis of NCC

Twenty four hour NCC was analyzed using a two-way ANOVA in which pCO_2_ (ambient and high), and sampling period (incubations 1–4 throughout the whole experiment) were fixed factors, and 24-h NCC was the response variable. Each sampling period consisted of 3 consecutive days that were treated as statistical replicates. The effect of time (incubation day) on the difference of 24-h NCC between ambient and high pCO_2_ was tested using a Model I least squares linear regression, in which a significant slope indicated that the treatment effect (i.e., the difference in 24-h NCC between ambient and high pCO_2_) changed over time. Separate regressions were completed for daytime and nighttime NCC in order to distinguish between the effects of OA on NCC during the day and night. All analyses were performed in R.

#### Analysis of NCP

NCP and night community respiration (R) was analyzed in a similar way to NCC using a two-way ANOVA in which pCO_2_ (ambient and high), and time (incubations 1–4 throughout each day) were fixed factors, and 12-h NCP or R was the response variable. Similar to NCC, each sampling period consisted of 3 consecutive days that were treated as statistical replicates.

#### Relationship between NCP and NCC

A change in community function (*sensu*^38^) was evaluated from the relative scaling of calcification as a function of productivity, with decreases in this quotient generally representing a degradation of the reef, leading to increased primary producers, and decreased calcifiers. The relationship between NCP and NCC was calculated as the slope of the latter on the former (expressed as change in mmol CaCO_3_ per mmol O_2_) over the 21-d incubation, in which hourly NCP was regressed on hourly NCC using Model II regression^31^. The slope, elevation, and corresponding error (95% CI) for each of the flumes was calculated using a Major Axis (MA) approach, in which error on both x- and y-axes are accounted for^39^. Significance of differing slopes between the high and ambient CO_2_ treatment were evaluated using a Bartlett-corrected likelihood ratio statistic, and the p-value was calculated assuming a chi-squared distribution with 1 degree of freedom^39^. Difference in elevation (y-intercepts) between treatment groups was evaluated using a Wald statistic that tested for no difference among the y-axis intercepts, and similar to slope significance, this statistic was evaluated using a p-value assuming a chi-squared distribution with 1 degree of freedom^39^. This analysis was performed on an aggregation of all sampling points within the 21-d incubation (4 time points, 3 days per time point, 4 samples per day = 48 paired NCP/NCC measurements per flume), and analyzed using a model II linear regression with the package smatr in R.

## Supplementary information

Supplementary Figures and Tables for Manuscript
