# Supplementary material for: Ocean acidification effects on in situ coral reef metabolism
Source: Sci Rep. 2019 Aug 19;9:12067. doi: 10.1038/s41598-019-48407-7 (PMC6700128; doi:10.1038/s41598-019-48407-7)
Supplement: Supplementary file 1 — Supplementary Figures and Tables for Manuscript [file 41598_2019_48407_MOESM1_ESM.docx]

**ELECTRONIC SUPPLEMENTARY MATERIAL**

**Ocean acidification effects on *in situ* coral reef metabolism**

Steve S. Doo, Peter J. Edmunds, Robert C. Carpenter


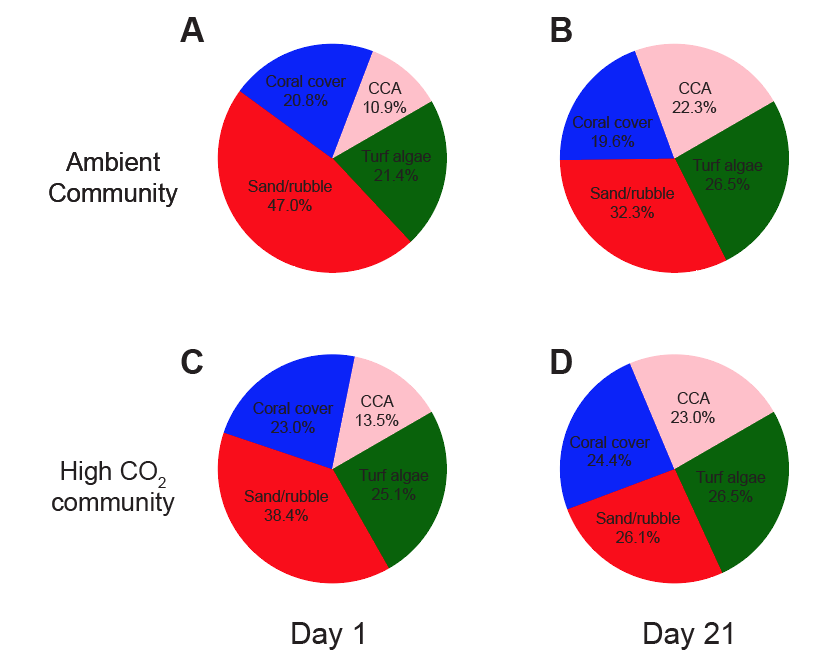


**Figure S1.** Pie charts showing benthic community structure in the plots of back reef community at ambient pCO_2_ **A.** on Day 1 (Initial), and **B.** Day 21 (Final), and elevated pCO_2_ on **C.** Day 1 (Initial), and **D.** Day 21 (Final). Each community was enclosed (2.5 m^2^ each) in separate flumes and incubated under high and ambient pCO_2_ for 21-d in May 2018. Percentage cover is based on analysis of planar images using 800 randomly placed dots for each image and manual annotation of component groups using CoralNet software^1^.


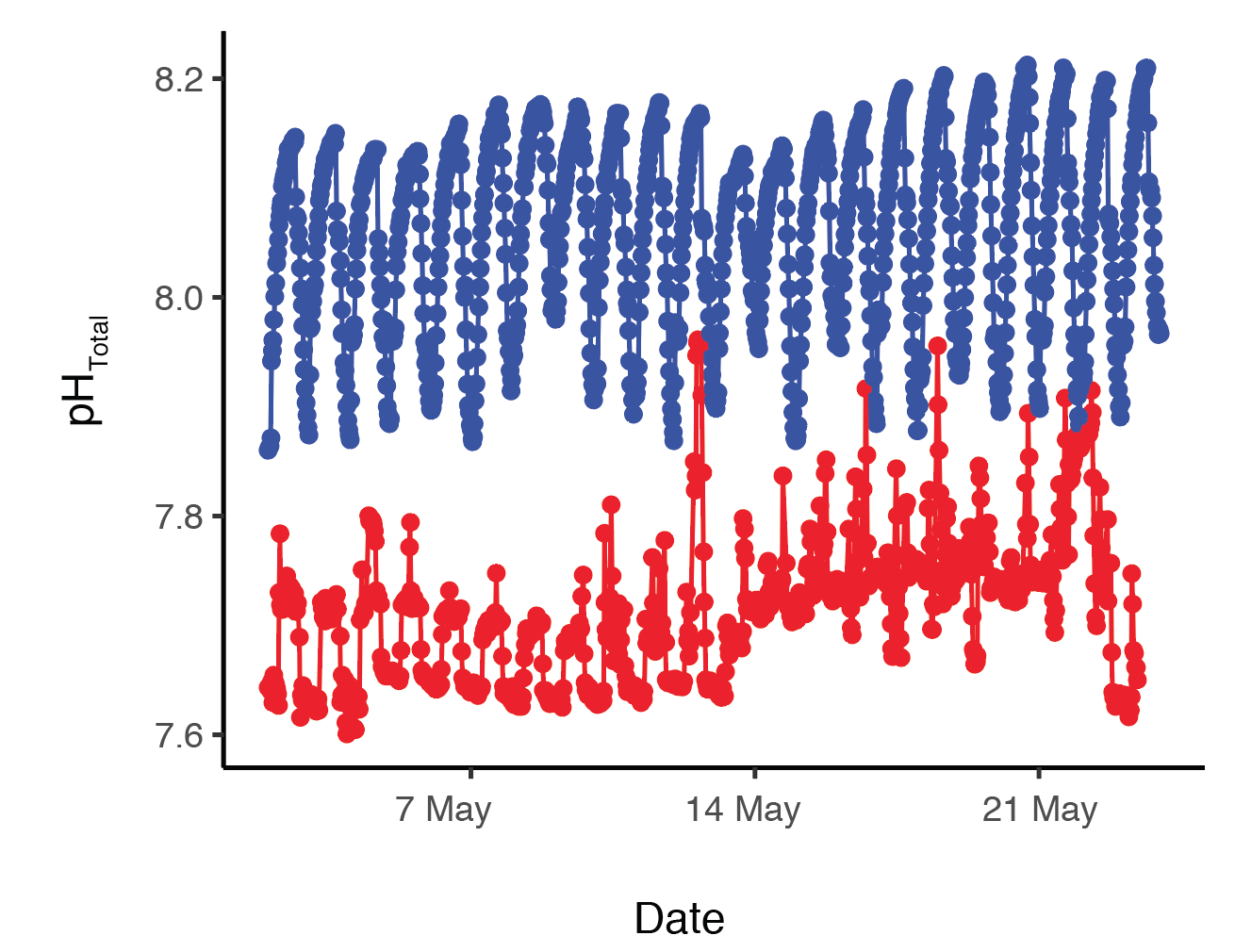
 **Figure S2.** Measurements of pH_Total_ in the ambient community (blue), and high CO_2_ community (red). The pCO_2_ for the high CO_2_ community was adjusted with a pH-dosing system in which pure CO_2_ was injected into the flume, while no modification of seawater in the ambient community was performed (other than hourly replenishment of seawater). Values of pH_Total_ within the flumes were recorded every 30 min, and measured with a SeaFET pH sensor (Durafet ® pH sensor).

**
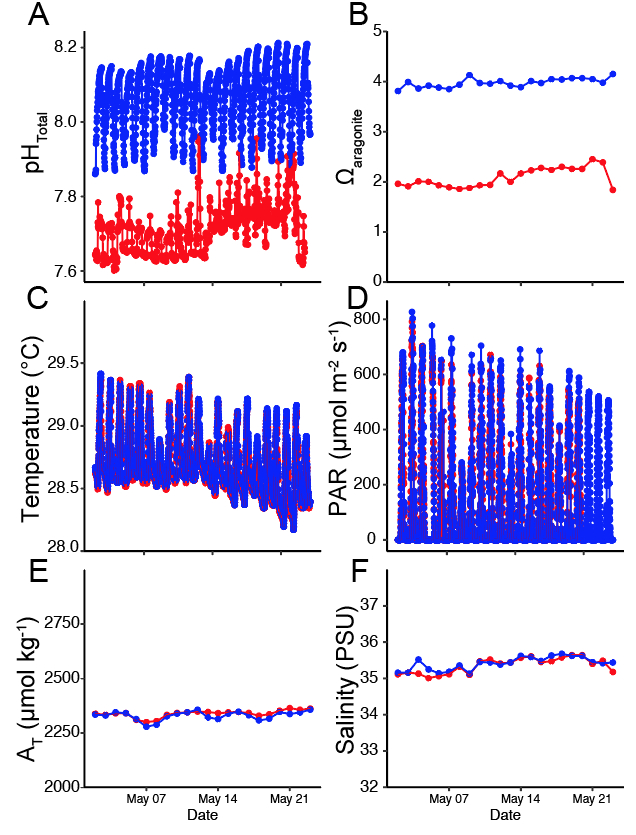
**

**Figure S3.** Physicochemical conditions during the 21-d experiment in the ambient (blue) and high pCO_2_ (red) treatments. (A) pH (measured every 30 min), (B) Aragonite saturation state (Ω_arag_; calculated daily), (C) Temperature (measured every 10 min), (D) Photosynthetically Active Radiation (PAR; measured every 10 min, (E) Total Alkalinity (A_T_ ; measured daily), and (F) Salinity (measured daily). All carbonate parameters were calculated using CO2SYS_v2.1.

**
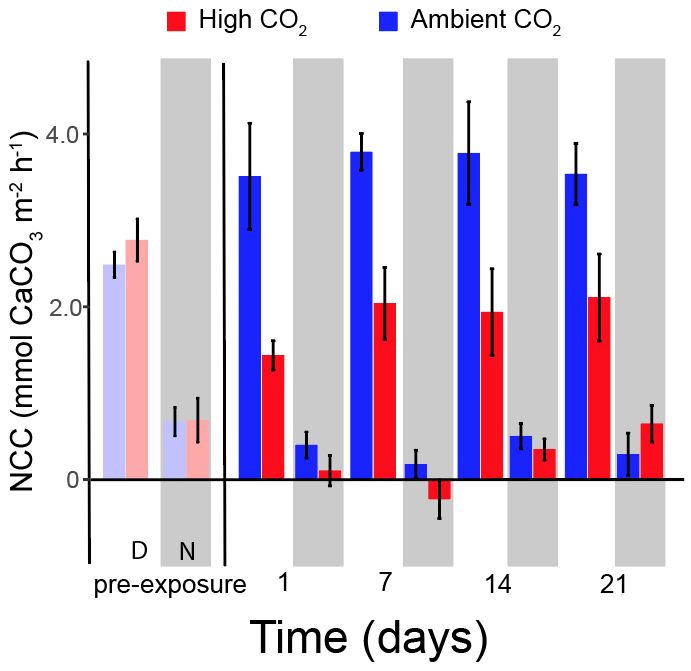
**

**Figure S4**. Mean (± S.E., n = 3 d) values of net community calcification (NCC) in the two back reef communities exposed to ambient (393 µatm; blue) or high (949 µatm; red) pCO_2_ over 21-d incubation, (light blue and light red shading represents communities of ambient and high CO_2_, respectively, prior to the initiation of treatment conditions). Measurements of daytime (unshaded) and nighttime (shaded grey) were averaged across the 3-d incubation period.

**
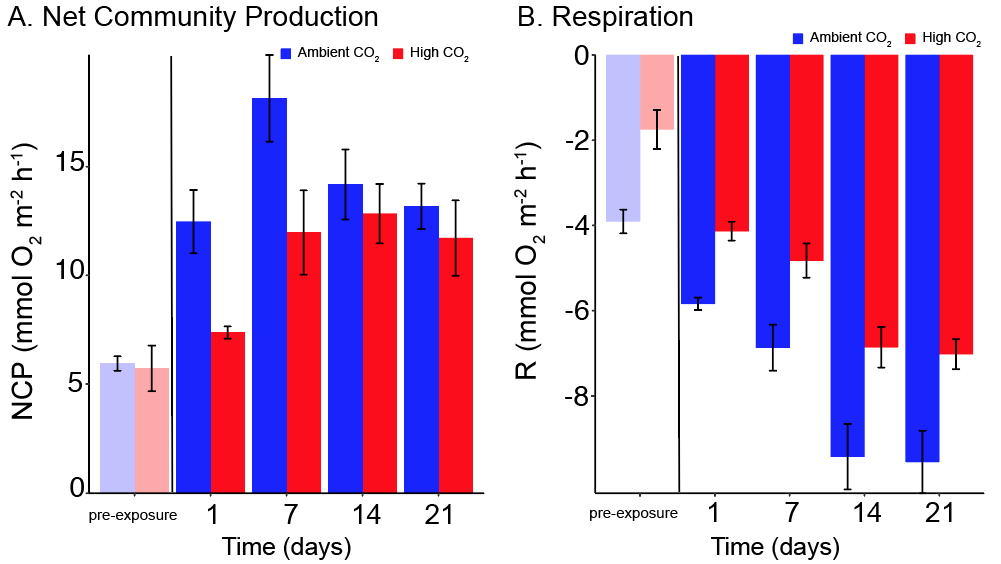
**

**Figure S5**. Mean (± S.E., n = 3 d) change in the two back reef communities exposed to ambient (393 µatm; blue) or high (949 µatm; red) pCO_2_ over 21-d incubation, (light blue and light red shading represents communities of ambient and high CO_2_, respectively, prior to the initiation of treatment conditions). (A) Daily integrated net community production (NCP), was measured three times during the day (average within day), and averaged over four blocks of three days beginning on day 1 (initial values) and ending on day 21 (final). **(B)** Net community respiration (R), was measured over one time interval overnight (sunset – sunrise), and averaged over the four blocks of three days, similar to NCP.

**Supplementary Table 1** Flow speeds within each flume (mean ± SE, cm s^-1^). Means represent an average of 27 randomly placed measurements per flume. Each measurement was calculated from an average flow speed of the summed vector averages for the X, Y, and Z velocities 60-s periods (sampled at 25 Hz). All measurements were performed with a Nortek Vectrino acoustic velocimeter.

| Flume | Flow Speed (cm s^-1^) |
| --- | --- |
| Ambient Community | 14.11 ± 0.37 (n = 27) |
| High CO_2_ Community | 14.79 ± 0.44 (n = 27) |

Supplementary Table 2. Statistical results tables for the analyses described in the methods. Post hoc analyses performed from ANOVA were Tukey HSD. Significant p-values are denoted in bold. Intercept and slope values are presented from ordinary least squares regressions ± 95% Confidence Intervals.

| **A. 24-hr NCC ANOVA Table** |  | | |  | | | |  | | | |  | | | |  | | | | | | | | | |  | | | | | |  |
| --- | --- | --- | --- | --- | --- | --- | --- | --- | --- | --- | --- | --- | --- | --- | --- | --- | --- | --- | --- | --- | --- | --- | --- | --- | --- | --- | --- | --- | --- | --- | --- | --- |
| Source | | df | | | SS | | | | MS | | | | F | | | | P | | | | | | | |  | | | | |  |  |  |
| Incubation Period | | 3 | | | 297.9 | | | | 99.3 | | | | 0.92 | | | | 0.450 | | | | | | | |  | | | | |  |  |  |
| CO_2_ | | 1 | | | 3088.9 | | | | 3088.9 | | | | 28.80 | | | | **<0.001** | | | | | | | |  | | | | |  |  |  |
| Incubation Period x CO_2_ | | 3 | | | 230.8 | | | | 76.9 | | | | 0.71 | | | | 0.556 | | | | | | | |  | | | | |  |  |  |
| Residuals | | 16 | | | 1715.9 | | | | 107.24 | | | |  | | | |  | | | | | | | |  | | | | |  |  |  |
| Total | | 23 | | | 5333.5 | | | |  | | | |  | | | |  | | | | | | | |  | | | | |  |  |  |
| **CO_2_:** High CO_2_ treatment < Ambient CO_2_ treatment | | | | | | | | | | | | | | | | | | | | | | | | | | | | | | | |  |
| **B. Daytime NCC_offset_ Regression** | | | |  | | | |  | | | |  | | | |  | | | | | | | | | |  | | | | | |  |
| Source | df | | | SS | | | | MS | | | | F | | | | P | | | | | | | | | |  | | | | | |  |
| Regression | 1 | | | 4.1 | | | | 4.1 | | | | 1.13 | | | | 0.295 | | | | | | | | | |  | | | | | |  |
| Residuals | 34 | | | 123.1 | | | | 3.6 | | | |  | | | |  | | | | | | | | | |  | | | | | |  |
| Total | 35 | | | 127.2 | | | |  | | | |  | | | |  | | | | | | | | | |  | | | | | |  |
| Intercept | 2.31 ± 0.61 mmol CaCO_3_ m^-2^ h^-1^ | | | | | | | | | | | | | | | | | | | | | | |  | | | | |  |  |  |  |
| Slope | -0.049 ± 0.47 mmol CaCO_3_ m^-2^ h^-1^ d^-1^ | | | | | | | | | | | | | | | | | | | | | | |  | | | | |  |  |  |  |
|  |  | | |  | | | |  | | | |  | | | |  | | | | | | | | | |  | | | | | |  |
| **C. Nighttime NCC_offset_ Regression** |  | | |  | | | |  | | | |  | | | |  | | | | | | | | | |  | | | | | |  |
| Source | df | | | SS | | | | MS | | | | F | | | | P | | | | | | | | | |  | | | | | |  |
| Regression | 1 | | | 0.8 | | | | 0.8 | | | | 5.57 | | | | **0.040** | | | | | | | | | |  | | | | | |  |
| Residuals | 10 | | | 1.4 | | | | 0.1 | | | |  | | | |  | | | | | | | | | |  | | | | | |  |
| Total | 11 | | | 2.2 | | | |  | | | |  | | | |  | | | | | | | | | |  | | | | | |  |
| Intercept | -0.53 ± 0.21 mmol CaCO_3_ m^-2^ h^-1^ | | | | | | | | | | | | | | | | | | | | |  | | | | |  |  |  |  |  |  |
| Slope | 0.038 ± 0.016 mmol CaCO_3_ m^-2^ h^-1^ d^-1^ | | | | | | | | | | | | | | | | |  | | |  | |  | | | | |  |  |  |  |  |
|  |  | | |  | | | |  | | | |  | | | |  | | | | | | | | | |  | | | | | |  |
| **D. NCP ANOVA Table** | | | |  | | | |  | | | |  | | | |  | | | | | | | | | |  | | | | | |  |
| Source | | | df | | | SS | | | | MS | | | | F | | | | | P | | | | | |  | | | | | | | |
| Incubation Period | | | 3 | | | 83.3 | | | | 27.8 | | | | 4.03 | | | | | **0.026** | | | | | |  | | | | | |  |  |
| CO_2_ | | | 1 | | | 73.7 | | | | 73.7 | | | | 10.70 | | | | | **0.005** | | | | | |  | | | | | | | |
| Incubation Period x CO_2_ | | | 3 | | | | 27.6 | | | | 9.2 | | | | 1.33 | | | | | 0.298 | | | | |  | | | | | | | |
| Residuals | | | 16 | | | 110.2 | | | | 6.9 | | | |  | | | | |  | | | | | |  | | | | | | | |
| Total | | | 23 | | | 294.8 | | | |  | | | |  | | | | |  | | | | | |  | | | | | | | |
| **Incubation Period:** Day 7 (A), Day 14 (AB), Day 21 (AB), Day 1 (B)  **CO_2_:** High CO_2_ treatment < Ambient CO_2_ treatment | | | | | | | | | | | | | | | | | | | | | | | | | | | | | | | |  |
|  | | | | | | | | | | | | | | | | | | | | | | | | | | | | | | | |  |
| **E. Net Community Respiration (R) ANOVA Table** | | | | | | | |  | | | |  | | | |  | | | | | | | | | |  | | | | | |  |
| Source | | | df | | | SS | | | | MS | | | | F | | | | | P | | | | | |  | | | | | | | |
| Incubation Period | | | 3 | | | 49.9 | | | | 16.6 | | | | 21.85 | | | | | **<0.001** | | | | | |  | | | | | |  |  |
| CO_2_ | | | 1 | | | 29.7 | | | | 29.7 | | | | 39.07 | | | | | **<0.001** | | | | | |  | | | | | | | |
| Incubation Period x CO_2_ | | | 3 | | | | 0.8 | | | | 0.3 | | | | 0.34 | | | | |  | | | | |  | | | | | | | |
| Residuals | | | 16 | | | 12.2 | | | | 0.8 | | | |  | | | | |  | | | | | |  | | | | | | | |
| Total | | | 23 | | | 124.1 | | | |  | | | |  | | | | |  | | | | | |  | | | | | | | |
| **Incubation Period:** Day 1 (A), Day 7 (A), Day 14 (B), Day 21 (B)  **CO_2_:** High CO_2_ treatment < Ambient CO_2_ treatment | | | | | | | | | | | | | | | | | | | | | | | | | | | | | | | |  |

Reference:

1. Beijbom, O., Edmunds, P. J., Kline, D. I., Mitchell, B. G. & Kriegman, D. Automated annotation of coral reef survey images. in 1170–1177 (IEEE, 2012). doi:10.1109/CVPR.2012.6247798
